# CXCL13/CXCR5 enhances sodium channel Nav1.8 current density via p38 MAP kinase in primary sensory neurons following inflammatory pain

**DOI:** 10.1038/srep34836

**Published:** 2016-10-06

**Authors:** Xiao-Bo Wu, De-Li Cao, Xin Zhang, Bao-Chun Jiang, Lin-Xia Zhao, Bin Qian, Yong-Jing Gao

**Affiliations:** 1 Pain Research Laboratory, Institute of Nautical Medicine, Jiangsu Key Laboratory of Inflammation and Molecular Drug Target, Nantong University, Nantong, Jiangsu 226019, China; 2 Department of Anesthesiology, The First People’s Hospital of Yancheng, Jiangsu 224005, China; 3 Co-innovation Center of Neuroregeneration, Nantong University, Nantong, Jiangsu 226001, China

## Abstract

CXCL13 is a B lymphocyte chemoattractant and activates CXCR5 receptor in the immune system. Here we investigated whether CXCL13/CXCR5 mediates inflammatory pain in dorsal root ganglia (DRG) and the underlying mechanisms. Peripheral injection of complete Freund’s Adjuvant (CFA) increased the expression of CXCL13 and CXCR5 in DRG neurons. In *Cxcr5*^−/−^ mice, CFA-induced pain hypersensitivity were attenuated. Whole-cell patch-clamp recording showed that the excitability of dissociated DRG neurons was increased after CFA injection or CXCL13 incubation from wild-type (WT) mice, but not from *Cxcr5*^−/−^ mice. Additionally, sodium channel Nav1.8 was co-expressed with CXCR5 in dissociated DRG neurons, and the increased neuronal excitability induced by CFA or CXCL13 was reduced by Nav1.8 blocker. Intrathecal injection of Nav1.8 blocker also attenuated intrathecal injection of CXCL13-induced pain hypersensitivity. Furthermore, CXCL13 increased Nav1.8 current density in DRG neurons, which was inhibited by p38 MAP kinase inhibitor. CFA and CXCL13 increased p38 phosphorylation in the DRG of WT mice but not *Cxcr5*^−/−^ mice. Finally, intrathecal p38 inhibitor alleviated CXCL13-induced pain hypersensitivity. Taken together, these results demonstrated that CXCL13, upregulated by peripheral inflammation, acts on CXCR5 on DRG neurons and activates p38, which increases Nav1.8 current density and further contributes to the maintenance of inflammatory pain.

Chemokines, which comprise a family of more than 50 family members, are chemotactic cytokines whose main function is to direct cell migration in the peripheral immune system^1^. A growing body of evidence shows that chemokines are also expressed in the central nervous system (CNS) to regulate the CNS function in both physiological and pathological conditions^1^,^2^,^3^. Recent studies have demonstrated that several chemokines (e.g., CCL2, CCL7, CXCL1, and CX3CL1) in the spinal cord and dorsal root ganglion (DRG) play critical roles in the pathogenesis of chronic pain induced by nerve injury or tissue inflammation^4^,^5^,^6^,^7^. Particularly, CXCL13, originally identified in stromal cells in B cell follicles, was recently found to be the most upregulated gene among detectable chemokines in the spinal cord after spinal nerve ligation (SNL)-induced neuropathic pain^8^. CXCR5, the sole receptor of CXCL13^3^,^9^, is expressed in spinal astrocytes and also increased after SNL. In addition, mice lacking *Cxcr5* show reduced neuropathic pain and decreased activation of spinal astrocytes and microglia^8^, suggesting the pivotal role of CXCL13/CXCR5 in the spinal cord in mediating glial activation and neuropathic pain.

Chronic inflammatory pain is a worldwide medical problem with low efficacy treatment options currently available^10^. Tissue inflammation is associated with short- and long-term changes in the excitability of sensory neurons in the DRG^11^. Evidence shows that several chemokines are expressed in the DRG and can regulate the neuronal excitability. For example, CXCL1 increases neuronal excitability of DRG neurons by increasing sodium currents and potassium currents^12^,^13^. Another chemokine, CCL2 was implicated in regulating sodium currents of DRG neurons^14^. CXCL13 mRNA was found to be increased in the DRG 14 days after local inflammation of DRG in rats^15^. Whether CXCL13 and CXCR5 in the DRG contribute to the pathogenesis of inflammatory pain and the implicated mechanisms are largely unknown.

In DRG neurons, voltage-gated sodium channels (VGSCs) are thought to play a critical role in the regulation of neuronal hyperexcitability that associated with nerve injury or peripheral inflammation^16^,^17^. Among the VGSCs, the tetrodotoxin-resistant (TTX-R) sodium channel Nav1.8 mostly contributes to the enhanced excitability and spontaneous ectopic discharges occurring in small- and medium-size DRG neurons in the chronic inflammation pain conditions^18^,^19^. Also, the hyperalgesia behavior evoked by inflammatory mediators was attenuated by blockade or knockout of Nav1.8 in animals^20^,^21^. *In vitro*, acute application of CXCL1^12^ or CCL2^14^,^22^ on cultured small sensory neurons increased the TTX-R Nav1.8 sodium current. Additionally, Nav1.8 within DRG neurons is a substrate for p38 mitogen-activated protein kinase (MAPK), and phosphorylation of Nav1.8 by p38 increases current density in these sensory neurons^23^. Whether CXCL13 could modulate the function of sodium channel Nav1.8 by p38 MAPK pathway remains to be investigated.

In the present study, we explored the role of CXCL13/CXCR5 in the DRG in inflammatory pain using the well-established complete Freund’s adjuvant (CFA) model. We examined the mRNA and protein expression for CXCL13 and CXCR5 in the DRG. We further investigated the direct role of CXCL13/CXCR5 in regulating neuronal excitability and Nav1.8 channel function in DRG neurons. Our results demonstrated that the expression of CXCL13 and CXCR5 was increased in the DRG after CFA-induced inflammatory pain, and CXCL13 may regulate Nav1.8 channel current density via CXCR5/p38 pathway.

## Results

### CFA increases CXCL13 expression in the DRG

**Figure 1:**
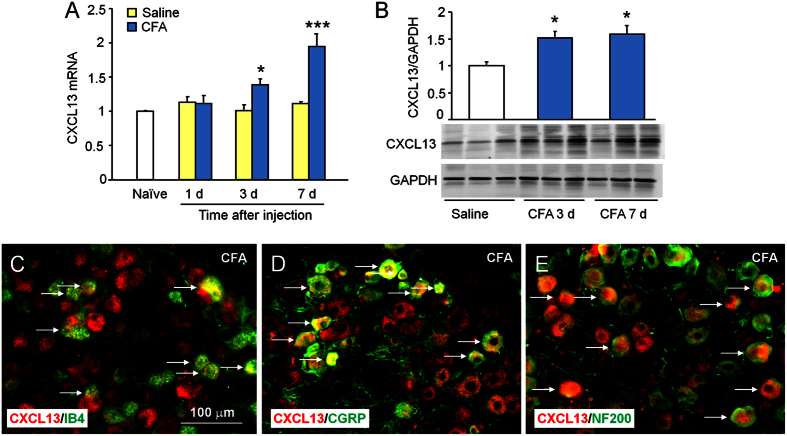
CXCL13 is increased in DRG neurons after CFA. (**A**) *Cxcl13* mRNA was increased at days 3 and 7 after CFA injection, compared with saline injection, Two-way ANOVA (Treatment, F_1,16_ = 20.12, P < 0.001; Time, F_2,16_ = 6.217, P < 0.01; Interaction, F_2,16_ = 6.646, P < 0.01). *P < 0.05, ***P < 0.001, post hoc Bonferroni test. (**B**) Western blot shows increased CXCL13 protein in the DRG after CFA. *P < 0.05, compared to saline. Student’s *t*-test. (**C–E**) Double staining of CXCL13 with IB4 (**C**), CGRP (**D**), and NF200 (**E**) in the DRG 3 days after CFA. Arrows indicate double-stained neurons.

Intraplantar injection of CFA induces robust mechanical allodynia and heat hyperalgesia, which started from 1 day and maintained for more than 7 days^24^. We first checked CXCL13 expression in the ipsilateral DRGs 1 day, 3 days, and 7 days after CFA or saline injection. *Cxcl13* mRNA was not changed at day 1, but significantly increased at days 3 and 7 in CFA-injected mice compared with saline-injected mice (Fig. 1A). The mRNA level did not significantly differ between naïve and saline-injected mice at all the time points (Fig. 1A). We further checked CXCL13 protein level by Western blot. As shown in Fig. 1B, CFA significantly increased CXCL13 protein at days 3 and 7 compared with saline, suggesting the possible involvement of CXCL13 in the DRG in CFA-induced inflammatory pain.

To detect the cellular distribution of CXCL13, we did double staining of CXCL13 with IB4, a marker of nonpeptidergic C-nociceptors (Fig. 1C); CGRP, a marker of peptidergic C-nociceptors (Fig. 1D); and NF200, a marker of A-fiber afferents (Fig. 1E). The results showed that CXCL13 was colocalized with all 3 markers, suggesting that CXCL13 is not specifically induced in one neurochemical cell type.

### CFA increases CXCR5 expression in the DRG

**Figure 2:**
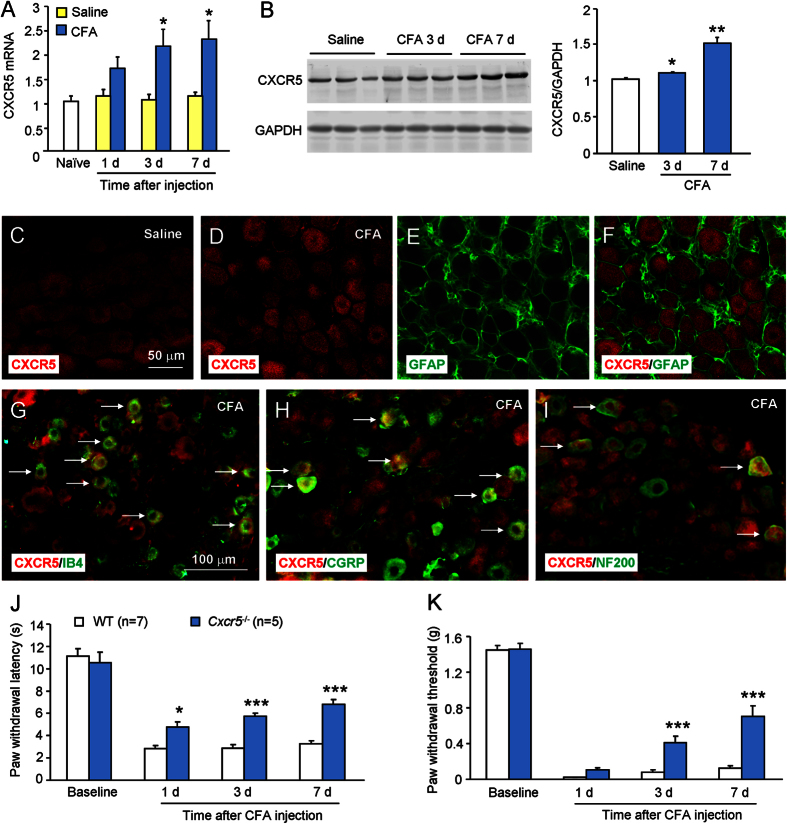
CXCR5 is increased in DRG neurons after CFA. (**A**) *Cxcr5* mRNA was increased at days 3 and 7 after CFA injection, compared with saline injection, Two-way ANOVA (Treatment, F_1,15_ = 17.18, P < 0.001; Time, F_2,15_ = 0.5842, P > 0.05; Interaction, F_2,15_ = 0.6964, P > 0.05). *P < 0.05, post hoc Bonferroni test. (**B**) Western blot shows increased CXCR5 protein in the DRG after CFA. *P < 0.05, **P < 0.01, compared to saline, Student’s *t*-test. (**C–F**) CXCR5 had low expression in the DRG of saline-treated mice (**C**), but was increased 3 days after CFA (**D**). Double staining of CXCR5 (**D**) with satellite marker GFAP (**E**). (**G–I**) Double staining of CXCR5 with IB4 (**G**), CGRP (**H**), and NF200 (**I**) in the DRG 3 days after CFA. Arrows indicate double-stained neurons. (**J,K**) CFA-induced heat hyperalgesia (**J**) and mechanical allodynia (**K**) were reduced in *Cxcr5* KO mice, compared with WT mice, Two-way repeated measures ANOVA (Heat hyperalgesia; Treatment, F_1,30_ = 39.5, P < 0.001; Time, F_3,30_ = 87.33, P < 0.001; Interaction, F_3,30_ = 6.706, P < 0.01. Mechanical allodynia; Treatment, F_1,30_ = 16.97, P < 0.01; Time, F_3,30_ = 553.6, P < 0.001; Interaction, F_3,30_ = 23.99, P < 0.001). *P < 0.05, ***P < 0.001, post hoc Bonferroni test.

We then examined CXCR5 expression in the DRG after CFA. As shown in Fig. 2A, CFA significantly increased *Cxcr5* mRNA expression at day 3 and day 7, but not at day 1. Saline injection did not change *Cxcr5* mRNA expression at days 1, 3, and 7. Furthermore, Western blot shows that CXCR5 protein was also increased 3 days and 7 days after CFA, compared with saline treatment (Fig. 2B). Immunofluorescence double staining showed that CXCR5 had low expression in the DRG of saline-treated mice (Fig. 2C), and was increased 3 days after CFA (Fig. 2D). Statistical data further showed that the percentage of CXCR5-positive cells was 15.9 ± 2.1% in naïve mice and increased to 43.9 ± 3.1% 3 days after CFA (P < 0.001, Student’s t-test). Double staining showed that CXCR5 was not colocalized with satellite cell marker GFAP (Fig. 2E,F), but was colocalized with IB4 (Fig. 2G) and CGRP (Fig. 2H), with a few in NF200-positive cells (Fig. 2I), indicating a major distribution of CXCR5 in small- and medium-sized DRG neurons.

We further check the role of CXCR5 in CFA-induced inflammatory pain. Pain behaviors were assessed after CFA injection in wild-type (WT) and *Cxcr5*^−/−^ (KO) mice. As shown in Fig. 2J,K, the baseline of paw withdrawal latency (Fig. 2J) or paw withdrawal threshold (Fig. 2K) was similar between WT and KO mice. However, CFA-induced heat hyperalgesia was significantly reduced in KO mice at 1 day, 3 days, and 7 days (Fig. 2J). CFA-induced mechanical allodynia was also significantly attenuated in KO mice at 3 days and 7 days (Fig. 2K). These data suggest that CXCR5 is involved in CFA-induced inflammatory pain.

### *Cxcr5* deletion reduces the enhanced neuronal excitability of DRG neurons induced by CFA or CXCL13

**Figure 3:**
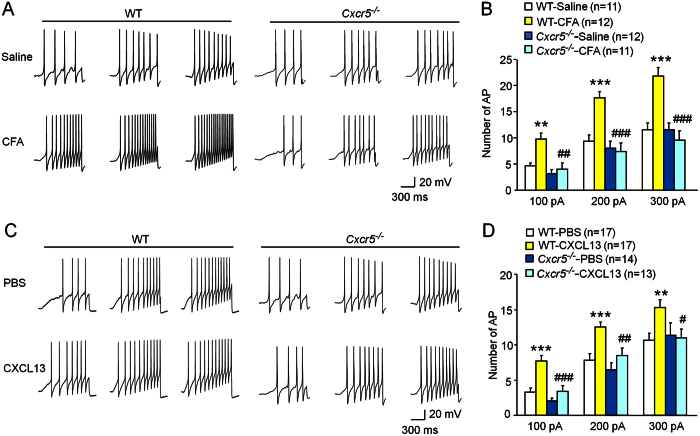
*Cxcr5* deletion reduces neuronal excitability of DRG neurons. (**A**) Examples of membrane potential responses evoked by 1000 ms ramp current injection of 100, 200, and 300 pA for neurons from saline-and CFA-treated wild type (WT) or *Cxcr5*^*−/−*^ mice. (**B**) Histogram shows an increase in numbers of action potential (AP) of DRG neurons from CFA-treated WT mice. Two-way repeated measures ANOVA (Treatment, F_3,84_ = 15.433, P < 0.001; Current, F_2,84_ = 123.976, P < 0.001; Interaction, F_6,84_ = 3.811, P < 0.05). **P < 0.01, ***P < 0.001, compared to WT-saline. ^##^P < 0.01, ^###^P < 0.001, compared to WT-CFA, post hoc Bonferroni test. (**C**) Examples of membrane potential responses evoked by 1000 ms current injection of 100, 200, and 300 pA for the PBS and CXCL13-treated neurons from WT or *Cxcr5*^*−/−*^ mice. (**D**) Histogram shows an increase in numbers of AP of DRG neurons from WT mice after CXCL13 treatment. Two-way repeated measures ANOVA (Treatment, F_3,114_ = 8.564, P < 0.001; Current, F_2,114_ = 161.529, P < 0.001; Interaction, F_6,114_ = 0.873, P > 0.05). **P < 0.01, ***P < 0.001, compared to WT-PBS. ^#^P < 0.05, ^##^P < 0.01, ^###^P < 0.001, compared to WT-CXCL13, post hoc Bonferroni test.

It was implicated that inflammatory pain is associated with increased excitability of nociceptive DRG neurons^11^. To check if CXCR5 is involved in the increased neuronal excitability after inflammation, we compared evoked action potentials (APs) of DRG neurons in WT and *Cxcr5* KO mice 3 days after CFA injection. The number of APs evoked by current stimulation was measured. In responding to 100 pA, 200 pA and 300 pA ramp current stimulation, the APs were dramatically increased in WT mice after CFA, but were not significantly changed in *Cxcr5* KO mice (Fig. 3A,B).

To further determine if CXCL13 is sufficient to increase the excitability of DRG neurons, we incubated DRG neurons from naïve mice with CXCL13 (100 ng/ml). In responding to 100 pA, 200 pA, and 300 pA ramp current stimulation, the APs were also increased in DRG neurons from WT mice, but not from *Cxcr5* KO mice (Fig. 3C,D). These results suggest that CXCL13/CXCR5 is involved in CFA-induced hyperexcitablity of DRG neurons.

### CFA and CXCL13 increase neuronal excitability via activation of Nav1.8 channel

**Figure 4:**
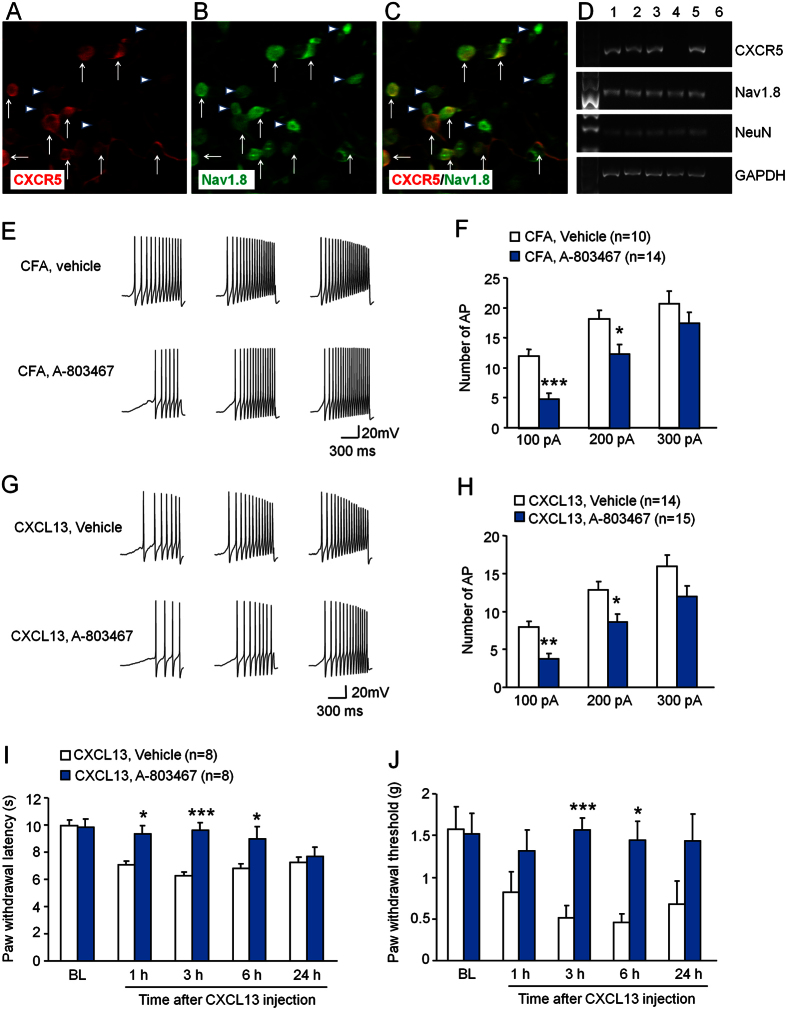
Nav1.8 channel contributes to CFA- and CXCL13-induced neuronal hyperexcitability and CXCL13-induced pain hypersensitivity. (**A–C**) Immunofluorescence staining shows the cellular distribution of CXCR5 (**A**) and Nav1.8 (**B**) in acutely dissociated DRG neurons from naïve mice. (**D**) Single-cell RT-PCR showing colocalization of CXCR5 with Nav1.8. No. 6 is negative control (bath solution). (**E**) Examples of membrane potential traces evoked by current injection in neurons dissected 3 days after CFA injection and pre-treated with vehicle (DMSO) or A-803467. (**F**) Histogram shows that A-803467 reduced the number of AP, compared to vehicle treatment. Two-way repeated measures ANOVA (Treatment, F_1,44_ = 6.966, P < 0.05; Current, F_2,44_ = 92.581, P < 0.001; Interaction, F_2,44_ = 3.076, P > 0.05). *P < 0.05, ***P < 0.001, post hoc Bonferroni test. (**G**) Examples of membrane potential traces evoked by current injection in neurons pretreated with vehicle (DMSO) or A803467 30 min before CXCL13 treatment. (**H**) Histogram shows that A-803467 reduced the number of AP, compared to vehicle treatment. Two-way repeated measures ANOVA (Treatment, F_3,54_ = 8.152, P < 0.01; Current, F_2,54_ = 80.840, P < 0.001; Interaction, F_2,54_ = 0.029, P > 0.05). *P < 0.01, **P < 0.01, post hoc Bonferroni test. (**I,J**) Intrathecal injection of A-803467 (10 nmol) attenuated intrathecal injection of CXCL13-induced heat hyperalgesia (**I**) and mechanical allodynia (**J**). Two-way repeated measures ANOVA (Heat hyperalgesia; Treatment, F_1,56_ = 25.03; P < 0.001; Time, F_4,36_ = 5.564, P < 0.001; Interaction, F_4,56_ = 3.276, P < 0.05. Mechanical allodynia; Treatment, F_1,56_ = 11.75; P < 0.01; Time, F_4,36_ = 2.943, P < 0.05; Interaction, F_4,56_ = 2.789, P < 0.05). *P < 0.05, ***P < 0.001 post hoc Bonferroni test. BL, baseline.

TTX-R sodium channel Nav1.8 plays a key role in the depolarization of AP in DRG neurons^25^,^26^,^27^. To investigate whether the increased APs in DRG neurons after CFA or CXCL13 incubation is induced through activation of Nav1.8, we first checked the distribution of CXCR5 and Nav1.8 in acutely dissociated DRG neurons. Immunofluorescence double staining showed that CXCR5 and Nav1.8 were highly, but not completely colocalized in small- and medium-sized DRG neurons (Fig. 4A–C). In addition, single-cell PCR further showed that 4 out of 5 neurons expressed CXCR5, and all of them expressed Nav1.8 (Fig. 4D), further supporting the colocalization of CXCR5 with Nav1.8 in DRG neurons.

We then checked the role of Nav1.8 on CFA- or CXCL13-induced hyperexcitablity of DRG neurons using A-803467, a potent and selective Nav1.8 sodium channel blocker^28^. DRG neurons from CFA-treated mice were incubated with vehicle (0.3% DMSO) or A-803467 (300 nM)^29^ for 30 min before neuronal excitability recording. As shown in Fig. 4E,F, the numbers of AP were reduced by A-803467 when the neurons were stimulated with 100 pA or 200 pA ramp current. However, the numbers of AP evoked by 300 pA ramp current stimulation were not significantly different between vehicle- and A-803467-treated neurons. We further determined whether A-803467 affects CXCL13-induced neuronal hyperexcitability. Our results revealed that treatment with A-803467 (300 nM) significantly reduced the APs evoked by current stimulation of 100 pA and 200 pA in DRG neurons incubated with CXCL13 (Fig. 4G,H). These data suggest that Nav1.8 is important in mediating CFA- and CXCL13-induced hyperexcitability of DRG neurons.

It was reported that blockade of Nav1.8 channel by A-803467 attenuated CFA-induced pain hypersensitivity^28^,^30^. We then asked if Nav1.8 channel activation contributes to CXCL13-induced pain hypersensitivity. A-803467 (10 nmol) was intrathecally injected 30 min before CXCL13 intrathecal injection. As shown in Fig. 4I,J, pretreatment with A-803467 blocked CXCL13-induced heat hyperalgesia at 1 h, 3 h, and 6 h (Fig. 4I). A-803467 also inhibited CXCL13-induced mechanical allodynia at 3 h and 6 h (Fig. 4J). These data suggest that Nav1.8 is important in mediating CXCL13-induced pain hypersensitivity.

### CXCL13 increases Nav1.8 current density in DRG neurons

**Figure 5:**
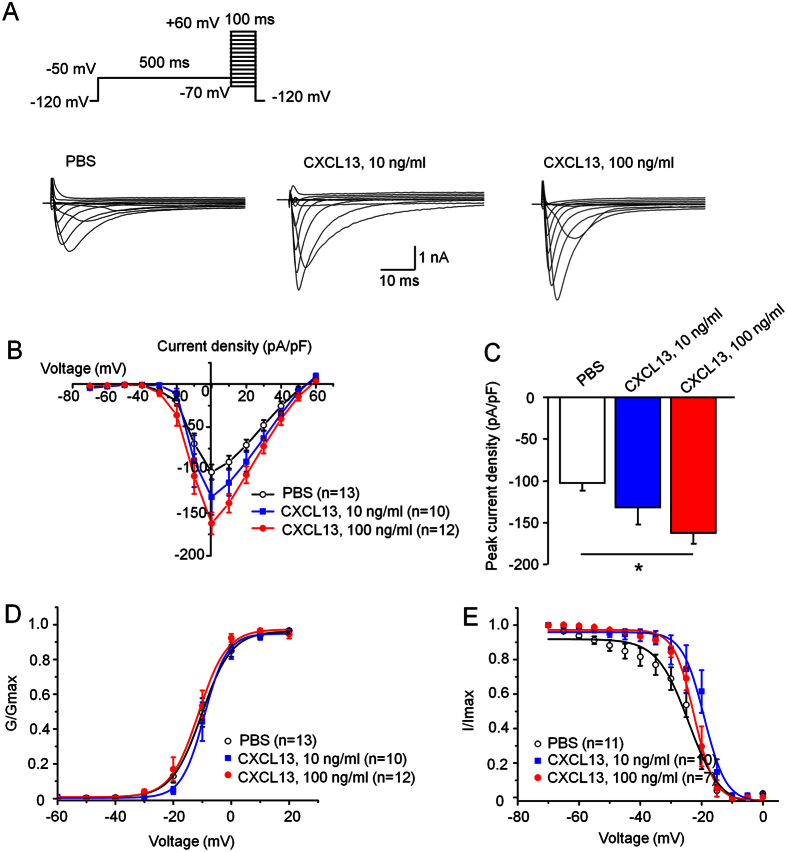
CXCL13 increases peak current density of TTX-R Nav1.8 in DRG neurons of naïve mice. (**A**) I–V curve family of currents was measured using the protocol with a 500 ms prepulse to -50 mV before each activating pulse. (**B**) Examples of voltage-clamp traces illustrating Nav1.8 current recorded following 30 min treatment with vehicle (PBS) or CXCL13 (10 ng/ml or 100 ng/ml). (**C**) The mean currents versus voltages (I–V) curves of Nav1.8 in DRG neurons. The peak current density of Nav1.8 was significantly enhanced in neurons pretreated with 100 ng/ml CXCL13. One-way ANOVA (F_2,32_ = 4.9346, P < 0.05). *P < 0.05, post hoc Bonferroni test. (**D**) Activation curves for Nav1.8 currents reveal no significant difference following CXCL13 treatment. (**E**) The steady-state inactivation curves for Nav1.8 currents also show no significant differences. P > 0.05, post hoc Bonferroni test.

We then checked if CXCL13 could directly modulate the TTX-resistant Nav1.8 sodium currents. The effects of CXCL13 on the density and kinetic properties of Nav1.8 currents were examined in acutely dissociated DRG neurons. The Nav1.8 current amplitude was significantly increased in CXCL13-treated neurons than that in vehicle-treated neurons (Fig. 5A). The mean peak current density of Nav1.8 in control neurons was 102.5 ± 8.9 pA/pF and increased to 131.6 ± 20.7 pA/pF and 162.3 ± 12.7 pA/pF after treatment with CXCL13 at 10 ng/ml and 100 ng/ml, respectively (Fig. 5B,C). We also tested the effects of CXCL13 treatment on activation and steady-state inactivation of Nav1.8. However, the voltage-dependent activation of Nav1.8 in neurons treated with PBS (V_1/2_ = −10.2 ± 0.1 mV; slope *k* = 5.1 ± 0.1 mV) was not significantly different from that with CXCL13 at 10 ng/ml (V_1/2_ = −9.3 ± 0.2 mV; *k* = 3.9 ± 0.2 m. P > 0.05) or 100 ng/ml (V_1/2_ = −11.3 ± 0.4 mV; slope *k* = 4.8 ± 0.4 mV. P > 0.05, One-way ANOVA. Fig. 5D). Similar to the activation curve, the steady-state inactivation parameter in PBS group (V_1/2inact_ = −24.2 ± 0.8 mV; *k* = 5.1 ± 0.7 mV) was also not significantly different from CXCL13 at the dose of 10 ng/ml (V_1/2inact_ = 19.2 ± 0.6 mV; *k* = 3.5 ± 0.5 mV) or 100 ng/ml (V_1/2inact_ = −22.5 ± 0.3 mV; *k* = 3.3 ± 0.3 mV. P > 0.05, One-way ANOVA, Fig. 5E). These data demonstrated that CXCL13 application mainly modulates current density but not kinetics of Nav1.8 in DRG sensory neurons in mice.

**Figure 6:**
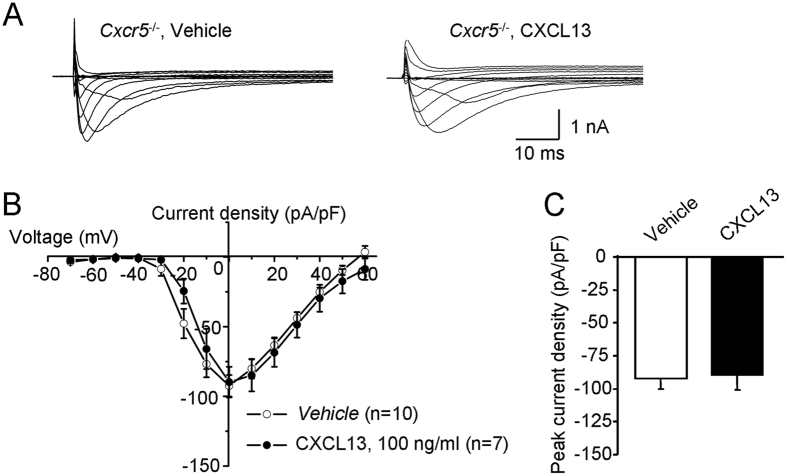
CXCL13 does not affect Nav1.8 current of DRG neurons from *Cxcr5*^*−/−*^ mice. (**A**) Examples of voltage-clamp traces illustrating Nav1.8 current recorded in PBS- or CXCL13 (100 ng/ml)-treated DRG neurons from *Cxcr5*^*−/−*^ mice. (**B**) The mean I–V curves of Nav1.8 from DRG neurons. (**C**) Histogram shows the mean peak current density of Nav1.8 in PBS- and CXCL13-treated DRG neurons. P > 0.05. Student’s *t*-test.

To further determine whether the increase in Nav1.8 current density after CXCL13 treatment is caused via CXCR5, we tested the Nav1.8 current in DRG neurons from *Cxcr5* KO mice (Fig. 6A). The Nav1.8 current amplitude was not significantly increased after CXCL13 treatment in DRG neurons from *Cxcr5* KO mice (P > 0.05, Student’s *t*-test, Fig. 6B). The mean peak current density of Nav1.8 was also comparable between CXCL13-treated and vehicle-treated neurons (P > 0.05, Student’s *t*-test, Fig. 6C).

### CXCL13 increases Nav1.8 current density via p38

**Figure 7:**
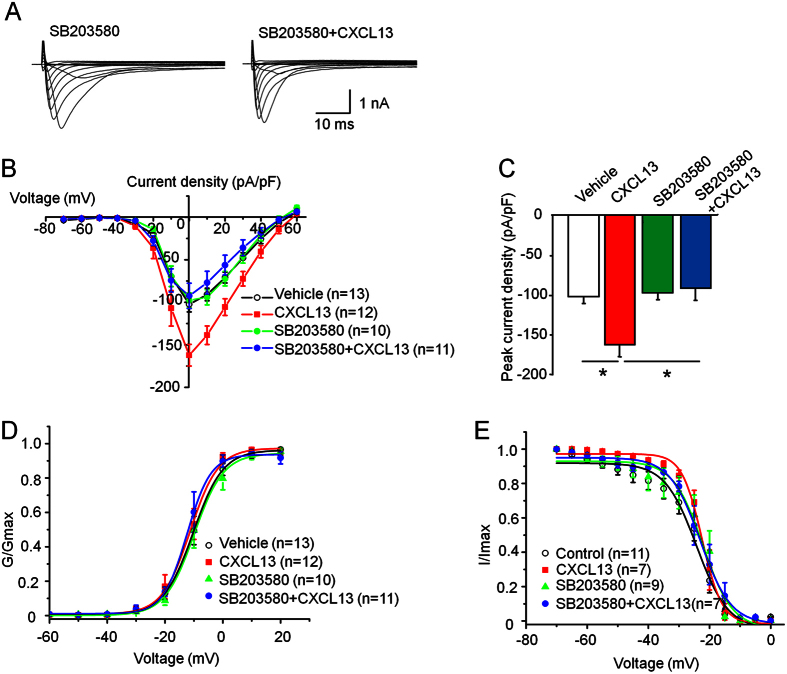
P38 is involved in CXCL13-mediated potentiation of Nav1.8 current density in DRG neurons. (**A**) Examples of voltage-clamp traces illustrating Nav1.8 current recorded in neurons pretreated with SB203580 (10 uM) or vehicle (0.1% DMSO). (**B**) The mean I–V curves of Nav1.8 from DRG neurons. (**C**) Histogram shows the specific p38 MAPK inhibitor SB203580 blocks CXCL13-mediated increase in Nav1.8 current density. One-way ANOVA (F_3,44_ = 7.4514, P < 0.001). (**D,E**) No significantly change was observed in the activation (**D**) or steady-state inactivation curves (**E**) for Nav1.8 in these treatments. P > 0.05. Student’s *t*-test.

P38 is an important intracellular kinase that contributes to both inflammatory pain and neuropathic pain in the DRG^31^. It was reported that activated p38 regulates neuronal excitability via activation of Nav1.8 channel in the DRG^32^. To determine whether p38 is involved in the increase of Nav1.8 current density after CXCL13 treatment, DRG neurons were pre-incubated with p38 MAP kinase inhibitor SB203580 (10 μM) for 30 min before CXCL13 treatment. Compared to vehicle, SB203580 alone did not change the current density of Nav1.8 (Fig. 7A). Pre-treatment of DRG neurons with SB203580 followed by a co-incubation with CXCL13 blocked the increase of Nav1.8 current density in DRG neurons, compared to neurons treated with CXCL13 alone (Fig. 7B,C). However, SB203580 did not affect the activation (SB203580: V_1/2_ = −10.0 ± 0.5 mV; slope *k* = 5.1 ± 0.4 mV; SB203580 + CXCL13: V_1/2_ = −12.4 ± 0.2 mV; slope *k* = 4.1 ± 0.2 mV. P > 0.05, Student’s *t*-test, Fig. 7D) or steady-state inactivation (SB203580: V_1/2inact_ = −19.1 ± 2.8 mV; slope *k* = 6.8 ± 1.5 mV; SB203580 + CXCL13: V_1/2inact_ = −22.0 ± 0.9 mV; slope *k* = 5.6 ± 0.6 mV. P > 0.05, Student’s *t*-test, Fig. 7E) of Nav1.8.

### p38 is involved in CXCL13/CXCR5-mediated pain hypersensitivity

**Figure 8:**
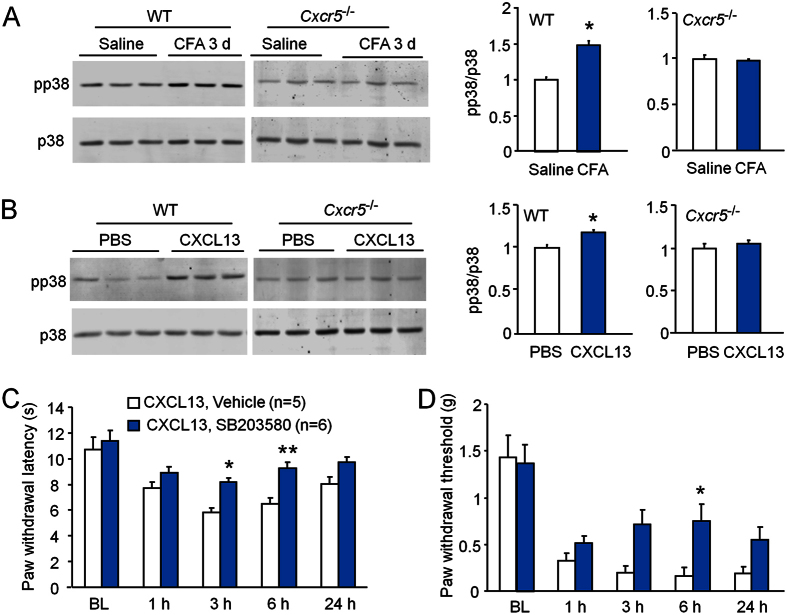
p38 is involved in CXCL13-induced pain hypersensitivity. (**A**) Western blot shows that pp38 expression in the DRG was increased 3 days after CFA in WT mice, but not in *Cxcr5* KO mice. *P < 0.05. Student’s *t*-test. (**B**) Western blot shows that intrathecal injection of CXCL13 increased pp38 expression in the DRG in WT mice, but not in KO mice. *P < 0.05. Student’s *t*-test. (**C,D**) Intrathecal injection of p38 inhibitor SB203580 attenuated CXCL13-induced heat hyperalgesia (**C**) and mechanical allodynia (**D**). Two-way repeated measures ANOVA (Heat hyperalgesia; Treatment, F_1,36_ = 35.64, P < 0.001; Time, F_4,36_ = 13.66, P < 0.001; Interaction, F_4,36_ = 1.062, P > 0.05. Mechanical allodynia; Treatment, F_1,36_ = 5.885, P < 0.05; Time, F_4,36_ = 24.29, P < 0.001; Interaction, F_4,36_ = 2.199, P > 0.05), *P < 0.05, **P < 0.01, post hoc Bonferroni test. BL, baseline.

To check if p38 is the downstream of CXCL13/CXCR5 pathway, we examined p38 activation in the DRG after CFA injection in WT and *Cxcr5* KO mice. Western blot showed that pp38 expression was markedly increased in the DRG of WT mice, but not of *Cxcr5* KO mice 3 days after CFA injection (Fig. 8A), suggesting that p38 may be a downstream of CXCR5 in the DRG.

Our previous study showed that intrathecal injection of CXCL13 induced both heat hyperalgesia and mechanical allodynia, which was dependent on CXCR5^8^. Here we further found that intrathecal injection of CXCL13 increased pp38 expression in the DRG in WT mice, but not in *Cxcr5* KO mice (Fig. 8B), suggesting that p38 in the DRG may be involved in CXCL13-induced pain hypersensitivity.

Finally, we checked if p38 inhibitor can affect intrathecal injection of CXCL13-induced pain. SB203580 (10 nmol) was intrathecally injected 30 min before CXCL13 injection. As shown in Fig. 8C, SB203580 attenuated CXCL13-induced heat hyperalgesia at 3 h and 6 h. The same treatment also attenuated CXCL13-induced mechanical allodynia at 6 h (Fig. 8D), indicating that p38 is involved in CXCL13-induced pain hypersensitivity.

## Discussion

Chemokine CXCL13 and CXCR5 is a recently reported chemokine pair that plays a pivotal role in mediating neuropathic pain in the spinal cord^8^. In the present study, we for the first time investigated the role of CXCL13/CXCR5 in the DRG in chronic inflammatory pain. We had the following new findings. First, we found that CXCL13 and CXCR5 were upregulated in the DRG neurons after CFA. CFA-induced pain hypersensitivity was reduced in *Cxcr5* deficient mice. Second, enhanced neuronal excitability of DRG neurons by CFA or CXCL13 was inhibited in mice lacking *Cxcr5*. Third, Nav1.8 current density was increased after CFA or treatment with CXCL13, and Nav1.8 mediated CXCL13-induced pain hypersensitivity. Finally, the increased current density of Nav1.8 by CXCL13 was mediated by p38 MAP kinase, which is also involved in CXCL13-induced pain. Collectively, our data revealed a novel role of DRG CXCL13/CXCR5 in mediating chronic inflammatory pain.

### Upregulation of CXCL13 and CXCR5 in the DRG and the involvement of CXCR5 in the pathogenesis of inflammatory pain

CXCL13, also known as B lymphocyte chemoattractant, was originally identified in stromal cells in B cell follicles to regulate homing of B cells and subsets of T cells^33^,^34^,^35^. Previous studies have shown that CXCL13 was induced in some microglia, macrophages, and endothelial cells in the CNS after infection^36^,^37^ or in infiltrating dendritic cells in EAE mice^38^,^39^. In the present study, CFA increased both the mRNA level and protein level of CXCL13 at 3 days and 7 days in the DRG. Immunostaining further showed a wide distribution of CXCL13 in the DRG neurons. Our recent data showed that CXCL13 was predominantly produced by spinal neurons after SNL^8^, indicating the neuronal expression of CXCL13 in both DRG and spinal cord, although we do not exclude a possible expression in satellite cells or macrophages in the DRG.

Chemokines and their receptors have been demonstrated to involve in chronic pain via distinct mechanisms at different anatomy locations^40^. For example, CX3CL1 and its receptor CX3CR1 regulate chronic pain via activation microglia in the spinal cord^41^, and via directly exciting primary nociceptive neurons in the DRG^42^. CXCL13 and CXCR5 are respectively expressed in neurons and astrocytes in the spinal cord and contribute to the pathogenesis of neuropathic pain through neuron-astroglial interaction^8^. Different from the astrocytic expression in the spinal cord, CXCR5 was expressed in the neurons of the DRG, with the main distribution in IB4^+^ and CGRP^+^ small- and medium-sized neurons, suggesting an autocrine/paracrine of CXCL13/CXCR5 signaling within the DRG. Furthermore, behavioral data showed that CFA-induced heat hyperalgesia and mechanical allodynia was markedly reduced in *Cxcr5* deficient mice at 3 days and 7 days. Taken with the upregulation of CXCL13 and CXCR5 in the DRG at 3 days and 7 days, but not at 1 day, these data suggest that CXCL13/CXCR5 in the DRG is involved in the maintenance, but not the early development of CFA-induced pain. As CXCL13 and CXCR5 are also expressed in the spinal cord^8^, spinal CXCL13/CXCR5 may also play a role in the pathogenesis of chronic inflammatory pain.

### CXCL13/CXCR5 regulates neuronal excitability of DRG sensory neurons via Nav1.8

VGSCs are very important for electrogenesis and nerve impulse conduction, and are believed to be involved in the pathogenesis of chronic pain in primary sensory neurons^16^. Nav1.8, which has a high activation threshold and slow inactivation kinetics, provides a significant contribution to action potential propagation within the sensory neurons of the DRGs^25^. In pathological pain conditions, not only the expression of Nav1.8 is altered^43^, the function of Nav1.8 channels is also increased^44^. Consistently, inhibition of Nav1.8 by antisense oligodeoxynucleotides or specific blockers reversed mechanical allodynia and thermal hyperalgesia after peripheral inflammation and nerve injury^28^,^45^. In addition, mice lacking Nav1.8 show a decreased sensitivity to mechanical stimuli and delayed development of thermal hyperalgesia^21^. Taken together, these findings strongly support a key role for Nav1.8 in regulating the properties of nociceptive neurons and nociceptive behaviors.

Evidence shows that Nav1.8 is regulated by a variety of inflammatory mediators, including TNF-α^46^, prostaglandin-E2^47^, endothelin-1^48^, and serotonin^49^. Recent studies also showed that chemokines, such as CCL2 and CXCL1 regulate neuronal excitability of DRG via increasing the function of Nav1.8^12^,^14^. Nav1.8 is mainly expressed in small- and medium-size C and A fibers in the DRG^14^,^50^. In this study, we found that CXCR5 was colocalized with Nav1.8. Moreover, the enhanced neuronal excitability by CFA or CXCL13 was abrogated in *Cxcr5* deficient mice and also reduced by Nav1.8 blocker in WT mice. CXCL13 also directly increased the density of Nav1.8 currents, although it did not affect kinetics of Nav1.8, which is different from the effect of CCL2 on Nav1.8^14^. Behavioral studies showed that A-803467, by i.v. injection, reduced mechanical allodynia in a variety of pain models including nerve injury-induced neuropathic pain and CFA-induced neuropathic pain in rats^28^. In addition, intrathecal injection of A-803467 also prevented capsaicin- or ischemia-induced mechanical allodynia^29^. We further showed that intrathecal injection of A-803467 attenuated CXCL13-induced mechanical allodynia and heat hyperalgesia. These results suggest that Nav1.8 is involved in CXCL13/CXCR5-mediated inflammatory pain in the DRG.

### The regulation of CXCL13/CXCR5 on Nav1.8 via p38 MAP kinase

It has been demonstrated that VGSCs can be modulated by receptors coupled to intracellular signaling molecules through the activation of cytoplasmic protein kinases. For example, prostaglandin E2 can act through protein kinase A and protein kinase C to increase the current density of Nav1.8^47^,^51^. PKC–NF-κB is involved in CCL2-induced elevation of Nav1.8 current density by promoting the phosphorylation of Nav1.8 and its expression^52^.

The p38 MAPK has been shown to be activated in DRG neurons after inflammation or nerve injury^53^,^54^,^55^. In addition, p38 is activated in small- and medium-sized DRG neurons after CFA^23^. Our study also showed increased expression of phosphorylated p38 in the DRG by Western blot. Moreover, intrathecal injection of CXCL13 induced p38 activation in WT mice, but not in *Cxcr5* deficient mice. It was reported that Nav1.8 and pp38 are colocalized in DRG neurons and p38 directly phosphorylates Nav1.8 to cause an increase in Nav1.8 current density^23^. We found that the increased Nav1.8 current density by CXCL13 was blocked by pretreatment with p38 inhibitor SB203580. Investigations also showed that p38 is involved in modulating the function of Nav1.8 in DRG neurons after treated with TNF-α^46^. Behavioral study showed that intrathecal SB203580 attenuated CXCL13-induced pain hypersensitivity, supporting that p38 is an important downstream of CXCL13/CXCR5 signaling which regulates the function of Nav1.8 channels and further contributes to inflammatory pain. As intrathecally injected CXCL13 and SB203580 also act on the spinal cord, SB203580 may also attenuate CXCL13-induced pain via inhibition of spinal p38 activation^56^.

ERK (extracellular signaling-activated kinase), another member of MAPKs, was shown to be activated in spinal astrocytes after intrathecal injection of CXCL13, which mediated astrocytes activation in the spinal cord during neuropathic pain^8^. In addition, we observed that ERK was also activated in the DRG after CXCL13 intrathecal injection (unpublished observation). We recently found that injection of CXCL13 into trigeminal ganglion (TG) induced ERK activation in the TG^44^, suggesting that ERK is also a downstream of CXCL13/CXCR5 in the DRG and TG. However, as p38 is exclusively expressed in microglia in the spinal cord^56^, whereas CXCR5 is not expressed in spinal microglia, CXCL13 may not directly activate p38 in the spinal cord, although the indirect effect cannot be ruled out. Meanwhile, how ERK mediates CXCL13/CXCR5-induced pain hypersensitivity in the DRG needs to be further investigated.

In summary, we explored the role of CXCL13/CXCR5 signaling in the DRG in inflammatory pain. Our results demonstrated that increased expression of CXCL13 and CXCR5 in DRG neurons regulates Nav1.8 current density via p38 MAP kinase and further contributes to the maintenance of inflammatory pain. Thus, the CXCL13/CXCR5 pathway may present a novel pharmacologic target for the treatment of chronic inflammatory pain.

## Materials and Methods

### Animals

Adult ICR mice (male, 8 weeks) were purchased from Experimental Animal Center of Nantong University. *Cxcr5*^−/−^ mice [B6.129S2 ^20^-Cxcr5^tm1Lipp/J^, stock number 006659] were purchased from the Jackson Laboratory and C57BL/6 wild-type mice were used as control. The animals were maintained on a 12:12 light–dark cycle at a room temperature of 22 ± 1 °C with free access to food and water. All animal procedures performed in this study were reviewed and approved by the Animal Care and Use Committee of Nantong University and were conducted in accordance with the guidelines of the International Association for the Study of Pain. Peripheral inflammation was induced by intraplantar injection of CFA (20 μl, Sigma, St Louis, MO) in the left hind paws^57^. The sham-treated animals were injected same volume of normal saline.

### Drugs and administration

Recombinant murine CXCL13 was purchased from PeproTech (Rocky Hills, NJ). SB203580, a highly specific, potent, and selective p38 MAP kinase inhibitor^58^, was purchased from Merck KGaA (Darmstadt, Germany). A-803467, a potent and selective Nav1.8 sodium channel blocker^20^, was purchased from Tocris (Minneapolis, MN). For intrathecal injection, spinal cord puncture was made with a 30 G needle between the L5 and L6 levels to deliver the reagents to the cerebral spinal fluid^59^.

### Real-time Quantitative PCR (qPCR)

The total RNA of the DRG was extracted using Trizol reagent (Invitrogen). One microgram of total RNA was reverse transcribed using an oligo(dT) primer according to the manufacturer’s protocol (Takara, Shiga, Japan). qPCR analysis of *Cxcl13* (Gene Bank Accession Number_NM_018866) and *Cxcr5* (Gene Bank Accession Number_NM_007551) was performed in the Real-time Detection System (Rotor-Gene 6000, Hamburg, Germany) by SYBR green I dye detection (Takara). The following primers were used: *Cxcl13* forward, 5′-GGC CAC GGT ATT CTG GAA GC-3′; *Cxcl13* reverse, 5′-ACC GAC AAC AGT TGA AAT CAC TC-3′; *Cxcr5* forward, 5′-TGG CCT TCTA CAG TAA CAG CA-3′; *Cxcr5* reverse, 5′-GCA TGA ATA CCG CCT TAA AGG AC-3′; *Gapdh* forward, 5′-GCT TGA AGG TGT TGC CCT CAG-3′; *Gapdh* reverse, 5′-AGA AGC CAG CGT TCA CCA GAC-3′. The PCR amplifications were performed at 95 °C for 30 s, followed by 40 cycles of thermal cycling at 95 °C for 5 s and 60 °C for 45 s. *Gapdh* was used as endogenous control to normalize differences. Melt curves were performed on completion of the cycles to ensure that nonspecific products were absent. Quantification was performed by normalizing Ct (cycle threshold) values with *Gapdh* Ct and analyzed with the 2^−ΔΔCT^ method^60^.

### Single-cell PCR

Single-cell RT-PCR for spinal neurons was performed as previously described^61^. Briefly, the contents of dissociated DRG neurons were harvested into patch pipettes with tip diameters of about 20 μm, gently put into reaction tubes containing Dnase I, then were kept at 37 °C for 40 min and followed by 80 °C for 10 min to remove contamination of genomic DNA. After adding reverse transcriptase (SuperScript III Platinum, Invitrogen), the samples were mixed gently and incubated at 50 °C for 50 min. The reaction was stopped by heating sample to 70 °C for 15 min. The cDNA products were used in gene-specific nested PCR. The sequences of the primers are shown in Table 1. The first round PCR was carried out using FastStart Universal SYBR Green Master (Roche, Switzerland). The following PCR conditions were used: 1 cycle of 3 min, 94 °C; 35 cycles of 15 s, 95 °C; 15 s, 60 °C; 1 cycle of 10 min, 72 °C. The second round of PCR was performed using 0.5 μl of the first PCR product as the template. The amplification regents and procedure for the inner primers was the same as that of the first round. A negative control was obtained from pipettes that did not have cell contents but were submerged in the bath solution. The PCR products were displayed on GelRed-stained agarose gels (3%).

### Western blot

Animals were transcardially perfused with PBS. The ipsilateral DRGs (L3-L5) were dissected and homogenized in a lysis buffer containing protease and phosphatase inhibitors (Sigma). Protein concentrations were determined by BCA Protein Assay (Pierce, Rockford, IL). Western blot was performed as previously described^62^. In brief, protein samples (30 μg) were separated on SDS–PAGE gel and transferred to nitrocellulose blots. The blots were blocked with 5% milk and incubated overnight at 4 °C with antibody against CXCL13 (Goat, 1:100, Santa Cruz, sc-8182), CXCR5 (rabbit, 1:100, Santa Cruz, sc-30029), pp38 (rabbit, 1:1000, Cell Signaling, #9215), p38 (rabbit, 1:1000, Cell Signaling, #9212), and GAPDH (mouse, 1:20000, Millipore, MAB374). These blots were further incubated with IRDye 800CW secondary antibodies for 2 h at room temperature, and captured by Odyssey Imaging System (LI-COR Bioscience, Lincoln, NE). Specific bands were evaluated by apparent molecular size. The intensity of the selected bands was analyzed using Image J software (NIH, Bethesda, MD).

### Immunohistochemistry

Animals were deeply anesthetized with isoflurane and perfused through the ascending aorta with PBS followed by 4% paraformaldehyde in 0.1 M PB. After the perfusion, the DRGs were removed and postfixed in the same fixative overnight. DRG sections (14 μm) were cut in a cryostat and processed for immunofluorescence as we described previously^5^. The sections were first blocked with 5% donkey serum for 2 h at room temperature, then incubated overnight at 4 °C with the following primary antibodies: CXCL13 (goat, 1:100, Santa Cruz, sc-8182), CXCR5 (rabbit, 1:100; Santa Cruz, sc-30029), glial fibrillary acidic protein (GFAP, mouse, 1:5000, Millipore, MAB360), peptidergic C-nociceptors marker, calcitonin gene–related peptide (CGRP) antibody (mouse, 1:5,000, Sigma-Aldrich, St. Louis, MO), nonpeptidergic C-nociceptors marker, IB4 (1:50; Sigma-Aldrich), and A fiber afferents marker, NF200 antibody (mouse, 1:500; Millipore). The sections were then incubated for 2 h at room temperature with Cy3- or Alexa 488-conjugated secondary antibodies (1:1000, Jackson ImmunoResearch, West Grove, PA). The stained sections were examined with a Leica fluorescence microscope, and images were captured with a CCD Spot camera.

### Behavioral analysis

Animals were habituated to the testing environment daily for at least two days before baseline testing. The room temperature and humidity remained stable for all experiments. For testing mechanical sensitivity, animals were put in boxes on an elevated metal mesh floor and allowed 30 min for habituation before examination. The plantar surface of each hindpaw was stimulated with a series of von Frey hairs with logarithmically incrementing stiffness (0.02–2.56 g, Stoelting, Wood Dale, IL), presented perpendicular to the plantar surface (2–3 s for each hair, 3 min interval between the tests). The 50% paw withdrawal threshold was determined using Dixon’s up-down method^63^. For testing heat sensitivity, animals were put in plastic boxes and allowed 30 min for habituation before examination. Heat sensitivity was tested by radiant heat using Hargreaves apparatus (IITC Life Science Inc., Woodland Hills, CA) and expressed as paw withdrawal latency. The test was repeated at least three times/animal allowing at least 5 min in between each test. The radiant heat intensity was adjusted so that basal PWL is between 10 and 14 s, with a cut-off of 18 s to prevent tissue damage^8^.

### Preparation of DRG neurons

Whole mount DRGs were isolated using a protocol as described previously^21^. Totally 53 mice were used for electrophysiological recording with 3–5 mice for each treatment. Briefly, the L4 and L5 DRGs were quickly removed from the vertebral column and placed in ice-cold oxygenated ACSF that contained the following (in mM): 125 NaCl, 3 KCl, 2.4 CaCl_2_, 1.2 MgCl_2_, 1.25 NaH_2_PO_4_, 26 NaHCO_3_, and 5 HEPES, pH 7.4. The connective tissue were carefully removed under a microscope, the ganglia were transferred to a 2 ml dissecting solution containing collagenase D (1.8 mg/ml, Roche, Basel, Switzerland) and trypsin (1 mg/ml, Amresco, OH) for 40 min at 37 °C. The ganglion was taken from the enzyme solution, washed and transferred into a holding chamber containing normal oxygenated ACSF in room temperature at least 1 hour before recording.

### Patch-clamp recordings from whole-mount DRG neurons

The patch-clamp recording experiments were performed at room temperature, and the ganglion was continuously perfused with ACSF saturated with 95% O_2_ and 5% CO_2_. Individual neurons were visualized under a stage-fixed upright IR-DIC microscope (BX51WI, Olympus) equipped with a 40× water-immersion objective. The patch pipettes were pulled from borosilicate glass capillary with filaments using a flaming micropipette pullers (P-97, Sutter Instruments), and had initial resistance 4~8 MΩ when filled with the internal pipette solution. Membrane voltage and current were amplified with a Multiclamp 700B amplifier (Molecular Devices). Data were filtered at 2 kHz and digitized at 10 kHz using a data acquisition interface (1440A, Molecular Devices). Liquid junction potential was corrected. The cell capacity transients were cancelled by the capacitive cancelation circuitry on the amplifier and series resistance was compensated for (>80%), and linear leak subtraction was subtracted digitally. The pClamp 10 software (Axon Instruments) was used for signal acquisition and analysis.

Small diameter (<25 μm) neurons were chosen for recording in the experiment. To measure the membrane potentials, the pipette solution was containing (in mM): 120 potassium gluconate, 20 KCl, 10 HEPES, 0.3 EGTA, 2 MgCl_2_, and 4 Na_2_ATP. The pH was adjusted to 7.3 with KOH, and osmolarity was 290~300 mOsm. For the study of neuronal excitability, resting potential (RP) and the action potential (AP) evoked by a series of ramp current stimulation (time: 1 sec; current intensity: 100 pA, 200 pA, 300 pA) were recorded.

For sodium currents recording, the voltage-clamp configuration was adopted. The ganglion was perfused at room temperature with the solution containing the following (in mM): 65 NMDG-Cl, 35 NaCl, 30 tetraethylammonium (TEA)-Cl, 0.1 CaCl_2_, 5 MgCl_2_, 0.1 CdCl_2_, 10 glucose, and 10 HEPES. pH was adjusted to 7.4 using NaOH and osmolarity was adjusted to 300 mOsm by glucose. TEA-Cl and CdCl_2_ were used to block voltage-gated K^+^ channels and Ca^2+^ channels, respectively. The pipette solution contained (in mM): 140 CsF, 10 NaCl, 5 EGTA, 2 Na_2_ATP, 1 MgCl_2_, 10 HEPES; pH adjusted to 7.2 with CsOH. Osmolarity was adjusted to 310 mOsm with glucose. In voltage-clamp experiments, TTX-R Nav1.8 currents were separated using moderate prepulse inactivation in the presence of TTX (0.3 uM) as described previously^14^,^64^. Briefly, test pulses were preceded 500 ms to −50 mV from a holding potential of −120 mV, and the voltage-current relationship of Nav1.8 currents were recorded in response to a series of depolarization steps that range from −70 mV to +60 mV in 10 mV increments with a time of 100 ms duration after the prepulse inactivation. I-V curves were generated by plotting current density as a function of testing potential. The current density was calculated by dividing the peak current amplitude by the cell capacitance (Cm) as directly read from the patch-clamp amplifier. The conductance (G) was calculated according to the equation G = I/(Vm − Vr), in which I is the peak current amplitude, Vm is the test potential, and Vr is the reversal potential for the current. Activation curves were fitted with the following Boltzmann distribution equation:


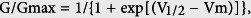


where Gmax is the maximum value for conductance, V_1/2_ is the potential at which activation is half-maximal, and k is the slope factor. The voltage-dependence of steady-state inactivation was measured by applying a 500 ms conditioning pre-pulses (ranges: −120 to −0 mV, increments: 5 mV), followed by a test pulse (−10 mV, 50 ms). The amplitude of I_Na_ was normalized to its respective maximum control value (I_max_) and plotted as a function of the conditional potential. The normalized curves were fitted by a Boltzmann distribution equation:


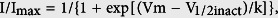


where Imax is the peak sodium current elicited after the most hyperpolarized prepulse, Vm is the preconditioning pulse potential, V_1/2inact_ is the potential at which I is half- Imax, and *k* is the slope factor.

For the drug treatment, CXCL13 was applied to the holding chamber for at least 30 min before experiments. Based on previous studies^5^,^29^, p38 inhibitor SB203580 or Nav1.8 blocker A-803467 was incubated for 30 min before co-treatment with CXCL13.

### Quantification and statistics

All data were expressed as mean ± SEM. The behavioral data and the action potentials were analyzed by two-way repeated measures ANOVA followed by Bonferroni test as the post-hoc multiple comparison analysis^8^. For western blot, the density of specific bands was measured with Image J. CXCL13 and CXCR5 levels were normalized to GAPDH, and pp38 levels were normalized to total p38^65^. Differences between groups were compared using one-way ANOVA followed by Bonferroni test. Student’s *t*-test was used if only 2 groups were applied. The criterion for statistical significance was P < 0.05.

## Additional Information

**How to cite this article**: Wu, X.-B. *et al*. CXCL13/CXCR5 enhances sodium channel Nav1.8 current density via p38 MAP kinase in primary sensory neurons following inflammatory pain. *Sci. Rep.* **6**, 34836; doi: 10.1038/srep34836 (2016).

## Figures and Tables

**Table 1:** Primers used for single cell RT-PCR.

Target gene (product length)	Outer primers	Inner primers	NCBI Reference Sequence
CXCR5 (445 bp, 274 bp)	5′-CTGTCTCAATCCCATGCTCTAC-3′5′-CCCTATGGCCAGGAAGAAATAA-3′	5′-CAGAGAATGCTACTTCCCTCAC-3′5′-GCTTAGCTTTAGCTGGTTAGGA-3′	NM_007551.2
Nav1.8 (316 bp, 203 bp)	5′-CATGACAGAGGAGCAGAAGAAG-3′5′-CCAGCCGTTGGTGAAGTAATA-3′	5′-CTTTGAATAAGTACCAGGGCTTC-3′5′-GAACATCTTCATCACACACTCG-3′	NM_001205321.1
NeuN (357 bp, 86 bp)	5′-AGACAGACAACCAGCAACTC-3′5′-CTGTTCCTACCACAGGGTTTAG-3′	5′-ACGATCGTAGAGGGACGGAA-3′5′-TTGGCATATGGGTTCCCAGG-3′	NM_001039167.1
GAPDH (367 bp, 313 bp)	5′-AGCCTCGTCCCGTAGACAAAA-3′5′-TTTTGGCTCCACCCCTTCA-3′	5′-TGAAGGTCGGTGTGAACGAATT-3′5′-GCTTTCTCCATGGTGGTGAAGA-3′	NM_008084.3
